# Effects of establishing infection control program with core components of World Health Organization on reducing the risk of residents’ infections and improving staff infection control competency in a nursing home

**DOI:** 10.1186/s13756-024-01492-4

**Published:** 2024-11-14

**Authors:** Min Hye Lee, Yu Mi Yi, Eun-Young Noh, Yeon-Hwan Park

**Affiliations:** 1 College of Nursing, Dong-A University, 32, Daesingongwon-ro, Seo-gu, Busan, 49201 Republic of Korea; 2 College of Nursing, Dong-Eui University, 176, Eomgwang-ro, Busanjin-gu, Busan, 47340 Republic of Korea; 3 Department of Nursing, Konkuk University, 268, Chungwon-daero, Chungju, 27478 Republic of Korea; 4 The Research Institute of Nursing Science, College of Nursing, Seoul National University, 103, Daehak- ro, Jongno-gu, Seoul, 03080 Republic of Korea

**Keywords:** Infection control, Nursing homes, Longitudinal studies, Resource-limited settings

## Abstract

**Background:**

Nursing homes (NHs) are high-risk facilities with limited infection control resources and residents susceptible to infectious diseases. The evidence regarding World Health Organization (WHO) core components in NHs is lacking. This study evaluates the effectiveness of establishing an infection prevention and control (IPC) program with WHO’s core components in an NH.

**Methods:**

The IPC program, encompassing evidence-based guidelines, education and training, surveillance, multimodal strategies, monitoring and feedback, workload and staffing considerations, and the built environment, was implemented in a 130-bed NH for one year. The effects were assessed based on the number of infections among residents, the level of knowledge, and the performance of infection control among staff. The risk of infection was analyzed across three phases: pre-implementation phase, implementation phase (6 and 12 months after intervention initiation), and sustainability phase (3, 6, and 12 months after intervention was finished). Staff data were analyzed before and after the intervention.

**Results:**

Analysis of 18,124 resident-days revealed that during the sustainability phase, the risk of respiratory tract infection was significantly lower than before intervention implementation (odds ratio [OR] 0.51, 95% CI 0.30–0.86, *p* = 0.012). Moreover, a significant improvement was observed in staff knowledge (*p* = 0.002) and performance (*p* < 0.001) after the intervention compared to before.

**Conclusions:**

WHO’s core components may have a potential effect on reducing healthcare-associated infections among residents and enhancing the infection control competency of staff in the NH with limited IPC resources.

**Supplementary Information:**

The online version contains supplementary material available at 10.1186/s13756-024-01492-4.

## Background

According to the United Nations [1], Korea is projected to experience one of the largest increases in the elderly population. Since the introduction of long-term care insurance in 2008 [2], the number of long-term care facilities (LTCFs), such as nursing homes (NHs), in South Korea has rapidly increased in response to the growing demand. An NH is a facility that provides care for older adults with chronic or gerontological diseases who have difficulty performing daily activities. Therefore, NHs house susceptible populations to infectious diseases due to aging, underlying medical conditions and chronic illnesses [3]. Facility and staff-related factors, such as the sharing of communal space, shortage of staff, and untrained staff, also hinder infection control in NHs [3]. These factors facilitate the transmission of infectious diseases and contribute to outbreaks of high-risk pathogens, such as severe acute respiratory syndrome coronavirus 2. In the United States, approximately 1–3 million severe infections occur annually in NHs and assisted-living facilities [4]. Furthermore, a cohort study showed that the mortality risk from the coronavirus disease of 2019 (COVID-19) among elderly residents in LTCFs is notably higher than among older adults living in the community [5]. The COVID-19 pandemic has highlighted several important lessons. First, the unclear boundaries of infectious diseases, which can extend beyond specific geographic areas or groups, allow the spread of infections within these facilities to be linked to community transmission. Second, effectively managing high-risk facilities that accommodate high-risk patients in communal living environments is essential, as infectious diseases do not impact all individuals equally. For these reasons, infection prevention and control (IPC) programs in NHs are crucial to ensuring the health and safety of residents and communities by preventing and reducing infectious disease transmission. Accordingly, the World Health Organization [6] proposed eight core components for the IPC in facilities: IPC programs, evidence-based guidelines, education and training, surveillance, multimodal strategies, monitoring and auditing of IPC practices and feedback, workload, staffing and bed occupancy, and built environment, materials and equipment [6].

Based on scientific evidence, expert consensus, and country experiences, the WHO core components for IPC are the foundation for strengthening effective national and facility-level programs. However, there is little data to support the efficacy of de novo implementation of IPC programs based on these WHO eight core components in NHs, particularly in South Korea. Aged care settings, such as NHs, are generally low-resourced, making it challenging to meet these requirements realistically. Low registered nurses (RN) staffing in NHs has been associated with an increased incidence of infectious diseases [7, 8], and RNs play a crucial role in infection control and prevention in NHs [9]. Nevertheless, over 70% of NHs in South Korea lack RNs [10]. While laws mandate staffing ratios per resident for RNs and certified nursing assistants, these ratios are often inadequate for effective infection control [11], and no regulations mandating designated personnel for infection control. Additionally, there is a lack of structures and resources for infection control, such as diagnostic and microbiological laboratory systems, infection control programs, and surveillance systems. The absence of an in-house diagnostic testing infrastructure delays the detection of infectious pathogens and disease. Consequently, due to these aforementioned challenges, NHs in Korea are generally considered under-resourced and understaffed [12].

The current study establishes an IPC program, including the WHO’s core components, in an NH in South Korea that did not previously have an IPC program. The primary objective was to evaluate the effectiveness of establishing an IPC program with WHO’s core components on the risk of healthcare-associated infection (HAI) (respiratory tract, urinary tract, skin and soft tissue, and gastrointestinal tract) among residents during a 2-year period and determine the role of the program in improving knowledge and compliance with infection control practices among nursing staff. Additionally, considering the lack of evidence on the long-term effects of IPC interventions in LTCFs [13], this study assesses the sustained effects over time. Hence, this study serves as a meaningful exemplar related to implementing an IPC program in an NH, offering valuable insights to complement the evidence regarding its effectiveness.

## Methods

### Study design and setting

This was a quasi-experimental before-and-after cohort study based on a cohort of all residents admitted to an NH in South Korea. The study site was purposively selected considering the feasibility and absence of any infection control intervention. The facility had a 130-bed capacity and approximately 70–80 workers. The NH did not have infection control guidelines, and no IPC program or professionals were dedicated to IPC activities before intervention. The institutional review board reviewed and approved this study (No. 1711/003–015, 1911/001–008). The intervention described below was implemented for 12 months, from January 1, 2018 to December 31, 2018 while data was collected between December 2017 and February 2020, after written informed consent was obtained from the workers and residents. Due to the health status of LTCF residents, individuals who had difficulty exercising autonomy required consent from proxies such as family members.

### Intervention

**Fig. 1:**
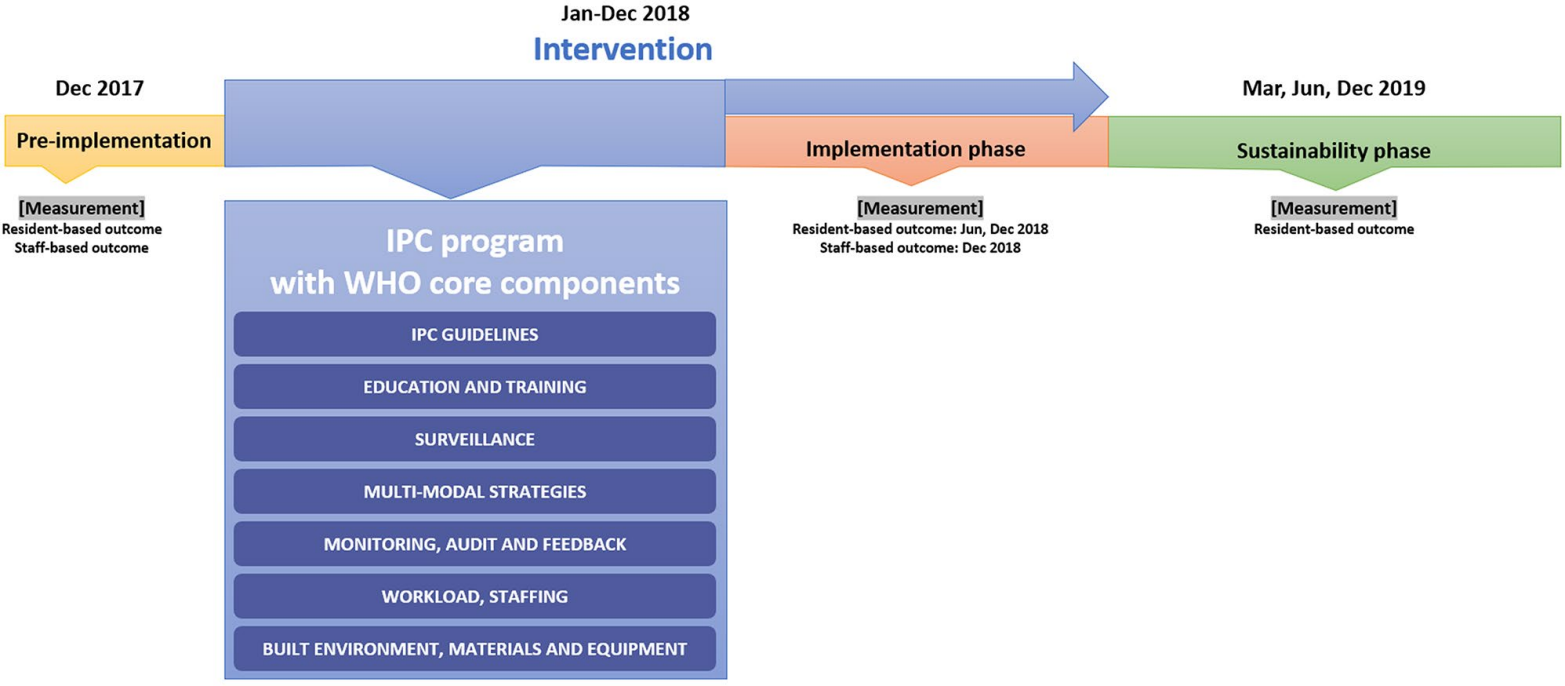
The timeline of this study. IPC = infection prevention and control

The multi-component IPC program was based on the IPC manual of the WHO [6] comprising evidence-based guidelines; education and training; surveillance; multi-modal strategies; monitoring and feedback; workload and staffing; and built environment, materials, and equipment (Fig. 1). The specific activities for each component are as follows:

### IPC guidelines

Based on the guideline development process of the Scottish Intercollegiate Guidelines Network [14], the guidelines were developed [15]. During the development, the infection control guidelines for LTCF provided by the Society for Healthcare Epidemiology of America (SHEA)/ Association for Professionals in Infection Control and Epidemiology (APIC) guideline for infection control in LTCF [16] were used as a foundation, along with guidelines and literature on infection control [17–19]. Printed guidelines were provided to the NH. The IPC practice algorithm [15], structuring the infection control procedures, was posted in units, including nursing stations and corridors, to facilitate application in daily practice.

### Education and training

Education specific to the IPC was implemented for all employees. Nursing staff, including nurses and certified caregivers, participated in 6 h of education delivered over 3 weeks, with one 2-hour session each week. The educational content included the characteristics of HAIs in LTCFs, standard precautions such as hand hygiene, transmission-based precautions, IPC guidelines, algorithms, nursing and assessment for infection prevention and control, occupational infection prevention and control (including immunization, medical evaluation, and management of exposure), visitor restrictions, environmental infection control, management of outbreaks, surveillance, and communication regarding infection-related information between healthcare facilities. An additional two-hour training session were provided after six months.

### Surveillance

Facility-based surveillance was performed by an external infection control nurse once weekly to detect HAI occurrences and outbreaks. The nurse had approximately 5 years of experience working as an infection control nurse at a tertiary hospital and played a role as part of the intervention in this study, but was not involved in data collection, analysis, or evaluation of the effectiveness of this intervention. The infection control nurse monitored residents for symptoms and signs of HAIs.

### Multi-modal strategies

Five elements of the WHO multi-modal strategy were applied, namely, system change, training and education, monitoring and feedback, reminders, and a culture of safety.

- System change: Before intervention, alcohol-based hand sanitizers were only placed at central stations of each unit, limiting access to hand sanitizers at the point of care. To address this issue, alcohol-based hand sanitizers, including pocket-sized hand rubs and paper towels, were supplied during the intervention. An educational kit for HH, such as a UV lamp for effective hand-washing demonstrations, was placed in the units.
- Training and education: In addition to the aforementioned education sessions for staff, the infection control nurse provided immediate on-site education and training to improve the staff’s infection control practices for 2 h per week.
- Monitoring and feedback: The infection control nurse monitored HAI occurrence and infection control practice by conducting direct observations to assess hand hygiene, proper glove use, environmental management, and compliance with IPC guidelines. Timley feedback was provided to staff on-site based on the results of these observations and the results of HH compliance were analyzed monthly and posted in units.
- Reminder and communication: Posters and checklists related to IPC activities were posted in each unit; the infection control nurse reminded the staff of proper IPC practices and infection control guidelines. The posters featured WHO’s hand hygiene indications, examples of nursing practices for each indication, proper techniques of hand hygiene, respiratory etiquette, visitor restrictions and the IPC practical algorithm.
- Culture of safety: An HH campaign was held for all employees at the NH in February 2018 to increase awareness of the importance of HH. Heads of nurses and certified caregivers were encouraged to participate in the intervention and lead their members actively. Employees with excellent IPC practices were selected monthly as role models, the list was posted in units, and incentives were awarded.

### Monitoring and feedback

The IPC practices of the NH were monitored weekly, and the infection control nurse provided timely feedback.

### Workload and staffing

The NH did not have dedicated infection control personnel before the intervention. Thus, we assigned the infection control nurse, who was hired by the research project, to the facility as a facilitator for implementing the IPC program. The infection control nurse visited the NH once weekly.

### Built environment, materials, and equipment

Products for HH, such as pocket-sized alcohol sanitizers and antimicrobial soaps containing chlorhexidine gluconate, were readily available at the point-of-care. Cleaning checklists were developed and used for environmental infection control on resident rooms, common areas, and bathrooms. These checklists offered guidelines for cleaning practices, detailing which environmental surfaces required cleaning, as well as specifying proper disinfectant use and verification of expiration dates.

### Measurements

The effect of the intervention was evaluated using resident- and staff-based outcomes. Resident-based outcomes were evaluated by the incidence of respiratory tract, urinary tract, GI, and SST infections—the most common infections in LTCFs [15, 20, 21]—defined by McGeer’s revised criteria [22], which were used for surveillance purposes. The study periods for the resident outcome evaluation were divided into three phases: ‘pre-implementation phase’ preceding the intervention, ‘implementation phase’ as the first evaluation of the intervention’s effect (with surveillance conducted at 6 and 12 months after intervention began), and ‘sustainability phase’ as the evaluation of long-term effects (with surveillance conducted at 3, 6, and 12 months after intervention was finished). The incidence of HAIs was calculated based on the number of new cases occurring over a one-month at each surveillance point, and the infection risk was analysed accordingly. A case that satisfied the criteria was classified as a definite case, while those that did not meet the criteria but in which the resident presented with symptoms of a possible infection were classified as probable cases. Infections included (1) definite and probable cases, and those (1) with no evidence of infection at the time of admission, (2) attributed to the NH, and (3) in residents on more than two calendar days after admission. The authors evaluated resident-based outcomes (MHL, YMY, and EYN). Initially, two authors independently carried out the surveillance, and then the results were reviewed and discussed with the third author to determine whether each case met the criteria.

Staff-based outcomes included the self-reported level of knowledge and compliance with infection control practices measured before and after the intervention. Knowledge of infection control practices was measured using a tool modified by Baek [23] according to the revised guidelines of the Hospital Infection Control Practices Advisory Committee [24], based on the tool designed by Suh and Oh [25]. This tool consists of a total of 29 items assessing standard precaution, HH, personal protective equipment, respiratory etiquette, placement of patient, environmental management, and sharp injury prevention. Each item was measured on a dichotomous scale: 1 point for correct answer and 0 points for wrong answer. A higher score indicated greater infection control knowledge. The reliability of the tool in this study was assessed based on the Kuder–Richardson 20 (KR-20) = 0.71. The performance of infection control practices was measured using a tool of Park and Lim [26], based on the infection control guidelines for elderly care facilities developed by Kim and Chun [27]. This tool comprised seven categories: HH, personal hygiene, disinfection, medication management, urinary tract infection management, respiratory infection management, and environmental management, and included 35 items rated on a 4-point Likert scale (1-never to 4-always). A higher score indicated a higher level of infection control performance. The reliability of this tool was Cronbach’s alpha = 0.96 in the developer’s study [26] and 0.96. in this study.

### Statistical analysis

Considering the characteristics of LTCFs, older adults who resided for ≥ 14 days were included in the final analysis. Data analysis was conducted using R 4.3.1 (R Foundation for Statistical Computing, Vienna, Austria; http://www.r-project.org/). For comparisons between groups (time points) in Tables 1, 3, and 4, one-way analysis of variance (ANOVA), independent sample *t*-tests, and chi-squared tests were performed. A Poisson regression model was used to predict the risk of infection based on the number of infections across the three phases of the study periods. However, a substantial proportion of the data had zero counts, making applying a standard Poisson model inappropriate. Therefore, the number of infections was modeled using a zero-inflated Poisson model with the ‘pscl’ package for analysis in Table 2. When conducting the analysis using the zero-inflated Poisson model, we adjusted for sex and age as covariates. We treated the number of resident days for each phase of study periods as an offset, representing at-risk days. All tests were conducted with a significance threshold set at *p* < 0.05.

A power analysis was performed using the G*Power 3.1.9 program [28] based on Poisson regression, with a focus on the odds ratio (OR) of resident outcomes. The analysis indicated that all resident outcomes achieved a power of 0.9 or higher, suggesting an adequate sample size, except for short-term total infection (OR = 1.06).

## Results

### Effectiveness of the intervention on resident-based outcomes

In total, 18,124 resident-day records were analyzed: 121 residents at pre-implementation phase, 249 during implementation phase, and 233 during the sustainability phase (Supplementary Fig. 1). The mean age of residents was 84.1 years, and 82.6% were female (Table 1). There were no significant differences in the characteristics of residents across the three phases.

The effectiveness on infection outcome are shown in Table 2. There was no significant difference in the likelihood of infections during implementation compared to pre-implementation (*p* = 0.615, *p* = 0.803 for respiratory and urinary tract infections, respectively). There was no difference in SST or GI infection risk between the implementation and pre-implementation phases. The risk of respiratory tract infection was significantly lower during the sustainability phase than at pre-implementation phase (OR 0.51, 95%CI 0.30–0.86, *p* = 0.012). There was no significant difference in the risk of infection during the sustainability phase compared to the pre-implementation phase for urinary tract infections and other infections.

**Table 1:** Resident characteristics across the three phases of outcome evaluation

Characteristic	Total (*n* = 603)	Pre-implementation phase (*n* = 121)	Implementation phase (*n* = 249)	Sustainability phase (*n* = 233)	*p*-value
Mean age (years)	84.1 ± 8.6	83.6 ± 8.8	84.0 ± 8.6	84.4 ± 8.5	0.667
Female sex	498 (82.6)	101 (83.5)	207 (83.1)	190 (81.5)	0.877
Length of stay per month (days)	30.1 ± 2.1	29.8 ± 1.6	30.1 ± 1.9	30.2 ± 2.6	0.213
Long-term care insurance rating*					0.473
1	121 (20.1)	28 (23.1)	47 (18.9)	46 (19.7)	
2	230 (38.1)	49 (40.5)	102 (41.0)	79 (33.9)	
3	213 (35.3)	37 (30.6)	87 (34.9)	89 (38.2)	
4	39 (6.5)	7 (5.8)	13 (5.2)	19 (8.2)	

*The long-term care insurance rating is determined by evaluating physical and mental functions to decide eligibility for long-term care insurance support. A lower rating indicates a higher dependency in performing activities of daily living

**Table 2:** Assessment of the intervention effectiveness on infection outcomes

Infection	Incidence of HAI						Effectiveness*			
	Pre-implementation phase		Implementation phase		Sustainability phase		Change after intervention (implementation phase vs. pre-implementation phase)		Long-term effect (sustainability phase vs. pre-implementation phase)	
	Rate (per 100 residents)	Density (per 1,000 resident days)	Rate (per 100 residents)	Density (per 1,000 resident days)	Rate (per 100 residents)	Density (per 1,000 resident days)	OR(95%CI)	*p*	OR(95%CI)	*p*
Respiratory tract	19.0	6.39	16.9	5.61	9.9	3.27	0.88(0.53, 1.46)	0.615	0.51(0.30, 0.86)	0.012†
Urinary tract	5.0	1.67	4.4	1.47	3.0	1.00	0.88(0.33, 2.38)	0.803	0.60(0.20, 1.78)	0.355
Skin and soft tissue	12.4	4.17	17.7	5.87	13.3	4.41	1.41(0.78, 2.53)	0.251	2.23(0.75, 6.60)	0.147
Gastrointestinal tract	1.7	0.56	3.6	1.20	3.4	1.14	2.16(0.45, 10.37)	0.335	2.05(0.42, 9.99)	0.376
Total	38.0	12.77	42.6	14.15	29.6	9.81	1.06(0.77, 1.48)	0.709	1.19(0.70, 2.03)	0.519

*adjusted for sex, age, and resident days, †*p* < 0.05, HAI: healthcare associated infection; OR: Odds ratio; CI: confidence interval

### Effectiveness of the intervention on staff-based outcomes

A total of 77 staff members in pre-test and 66 in post-test were included in the final analysis (Table 3). The mean age was 52.22 years on pre-test and 54.02 years on post-test, showing no significant difference. Most staff were female (pre-test: *n* = 74, 96.1%, post-test: *n* = 63, 95.5%), and 10–15% were nurses (pre-test: *n* = 8, 10.4%, post-test: *n* = 10, 15.2%). There was also no statistically significant difference in the time employed in the facility between the pre- and post-groups.

A significant increase was observed in the knowledge of infection control in post-test compared to pre-test groups (*p* = 0.002; Table 4). Regarding the sub-categories, the level of knowledge regarding standard precautions (*p* = 0.004), respiratory etiquette (*p* = 0.025), and resident placement (*p* = 0.006) was significantly higher post-test than pre-test. Moreover, the level of performance for infection control practices significantly increased compared to pre-test (*p* < 0.001). The level of all infection control practices improved in post-test, including HH (*p <* 0.001), urinary care (*p* = 0.003), respiratory care (*p <* 0.001), personal hygiene (*p* = 0.003), and environmental control (*p <* 0.001).

**Table 3:** Staff characteristics in pre- and post-test

Characteristics	Pre-test (*n* = 77)	Post-test (*n* = 66)	t or χ^2^	*p*
Age (year)	52.22 ± 8.41	54.02 ± 8.90	-1.24	0.218
Sex				
Female	74 (96.1)	63 (95.5)	0.037*	0.999
Male	3 (3.9)	3 (4.5)		
Education level (year)	13.18 ± 2.42	13.15 ± 2.26	0.08	0.939
Job			1.84*	0.809
Nurse	8 (10.4)	10 (15.2)		
Licensed caregiver	58 (75.3)	48 (72.7)		
Physical therapist	2 (2.6)	3 (4.5)		
Social worker	7 (9.1)	4 (6.1)		
Others	2 (2.6)	1 (1.5)		
Working Career (month)	76.43 ± 80.86†	93.77 ± 105.30	-1.11	0.270
Career in current facility				
< 1 year	17 (22.1)	15 (22.7)	4.97*	0.294
1–2 years	18 (23.4)	8 (12.1)		
2–3 years	6 (7.8)	11 (16.7)		
3–5 years	13 (16.9)	13 (19.7)		
≥ 5 years	23 (29.9)	19 (28.8)		

*Fisher’s exact test, †*n* = 76

**Table 4:** Intervention effectiveness on knowledge and performance of infection control practice among staff

Variable	Pre-test (*n* = 77)	Post-test (*n* = 66)	t	*p*
Knowledge of infection control practice	19.82 ± 3.25	21.26 ± 2.17	-3.150	0.002*
Standard precautions	2.34 ± 1.35	2.91 ± 1.00	-2.892	0.004*
Hand hygiene	3.12 ± 0.86	3.23 ± 0.74	-0.817	0.415
Personal protective equipment	7.97 ± 1.46	8.27 ± 1.20	-1.324	0.188
Environmental control	3.01 ± 0.84	3.11 ± 0.68	-0.722	0.471
Respiratory etiquette	1.77 ± 0.46	1.91 ± 0.29	-2.267	0.025*
Resident placement	1.61 ± 0.57	1.83 ± 0.38	-2.811	0.006*
**Variable**	**Pre-test** (***n*** **= 72)**	**Post-test** (***n*** **= 66)**	***t***	***P***
Performance of infection control practice	118.58 ± 23.54	133.59 ± 10.18	-4.931	< 0.001*
Hand hygiene	39.76 ± 7.83	46.14 ± 3.29	-6.327	< 0.001*
Personal hygiene	21.39 ± 2.97	22.73 ± 2.09	-3.081	0.003*
Urinary care	13.79 ± 4.16	15.44 ± 1.92	-3.030	0.003*
Respiratory care	19.58 ± 5.54	22.33 ± 2.75	-3.737	< 0.001*
Environmental control	24.06 ± 5.69	26.95 ± 2.14	-4.020	< 0.001*

*p < 0.005

## Discussion

This study assessed the effects of establishing an IPC program, based on WHO’s core components, in an NH setting with limited infection control resources on residents and staff. Recently, infection control in high-risk facilities, where resources are limited and vulnerable populations reside, has become a critical public health focus. Within the current study, the overall decrease in the risk of respiratory tract infections suggests a possible effect of de novo implementation of the IPC program in NHs.

This IPC program may have a long-term effect on reducing the risk of respiratory tract infections, which aligns with a systematic review of 17 studies that found that IPC programs employing education, monitoring, feedback, and the four to five components of a multi-modal strategy, demonstrate effectiveness in LTCFs [13]. The introduction and maintenance of the IPC program with WHO’s core components in low-resourced settings can ultimately contribute to the prevention of HAIs and enhance the provision of safe care to residents. The IPC program in this study does not have a vertical approach targeting specific pathogens; rather, it mainly focuses on reinforcing standard precautions and hygiene management. A horizontal approach might be more effective than a vertical intervention, particularly in LTCFs with poor access to laboratory tests and monthly physician visits [29, 30].

The present study demonstrates a significant improvement in the level of knowledge and performance of infection control practices of staff after the intervention, indicating the successful promotion of behavioral change through the multi-component IPC program. In the case of HH, there was no significant difference in the level of knowledge, but the performance significantly improved. This suggests that because HH is a fundamental practice; the level of knowledge was already relatively high before the intervention. Therefore, it is interpreted that the intervention facilitated the application of knowledge to practice, leading to actual behavior changes. A previously reported study from our IPC program, a consistent improvement in staff HH compliance has also been observed as a process indicator [31], supporting this interpretation. This study offers insights into several key factors that can contribute to sustained adherence of staff for continuous improvement [32]. Firstly, the current study implemented evidence-based IPC guidelines and multi-modal interventions for 12 months to improve staff adherence. The year-long efforts of this study have contributed to establishing an organizational culture for infection control within the NH. These long-term efforts may be required to enhance staff performance in facilities with limited resources. Secondly, the multi-modal strategy appears to have been significant. Incentives and role models, as key elements of the culture of safety, help encourage institutional staff and administrators to actively participate in the IPC program and exhibit leadership. Indeed, previous studies have reported that effective leadership and administrative engagement improve staff performance [33, 34]. Furthermore, considering the general staff shortage and staff overload, previous studies have demonstrated that strategies including ongoing training and on-site education, such as those applied in our study, are beneficial to achieve behavioral changes in staff [35, 36]. Finally, the placement of the infection control nurse has been crucial in addressing the staff shortage and absence of dedicated infection control personnel. In low-resource settings, a substantial workload is a major obstacle to implementing effective infection control [37]. In the United States, ~ 10% of LTCF staff are RNs [38], generally lacking training and education regarding infection control [39]. Similarly, in South Korea, approximately 70% of NHs lack RNs, and no regulations mandating dedicated infection control personnel or structured IPC programs for NHs [10]. Therefore, we included the infection control nurse as an intervention element, drawing inspiration from the concept of an ‘infection control link nurse (ICLN)’ [40]. Since infection control nurses improved compliance on guidance and infection control practices, reducing infection risks, it would be worth reinforcing external IPC support in NHs with limited human resources by deploying ICLN nurses at the district level [41].

This study did not find a significant reduction in the risk of HAIs other than respiratory tract infections. This may be due to various challenges. First, due to the nature of NH, private rooms are limited; thus, the shared living spaces may have influenced nosocomial transmission. In particular, all residents share bathrooms, which could act as pathogen reservoirs related to GI and SST infections [42]. It is possible that the absence of risk reductions in GI and SST infections could be attributed to these factors. Moreover, considering that GI infections are the most prevalent type of infection in LTCFs, also in the context of outbreaks [43], the absence of GI infection outbreaks during the intervention might indicate a positive effect of the IPC program. Second, barriers related to human resources have impeded effective infection control. In fact, staff turnover in LTCFs is reportedly ~ 50% [44]. Similarly, in this study, the frequent nursing staff turnover acted as a barrier to the IPC program. Finally, diagnosing infections may be challenging in the NH population [45]. The case definition applied in this study was used for surveillance purposes and is meaningful only for identifying the possibility of an event, not for diagnosing an actual infection. In particular, the older residents in NHs may exhibit atypical presentations that may not meet the surveillance definition, which does not indicate the absence of infection. Additionally, the absence of microbiological laboratory capacity in the NH might have influenced the surveillance process.

To the best of our knowledge, this is the first study to confirm the positive effects of a de novo implementation of an IPC program applying all core components of the WHO on both infection risk of residents as well on knowledge and performance on infection control among staff, and to evaluate its long-term effects. Despite these strengths, this study has several limitations. First, the absence of a control group leads to the impact of exogenous variables, such as maturation, necessitating caution in interpreting the intervention effects. Furthermore, given the challenges of diagnostic testing, the number of HAIs identified through surveillance may have been underestimated. This low number of cases could have affected the statistical power to detect the effectiveness of the intervention. The analysis of short-term effect on total infection showed a power of approximately 0.7, suggesting that this specific analysis may be underpowered. Second, blinding was not performed when measuring the effects on infectious diseases; however, this did not likely impact the results given the increased number of certain infectious diseases. Third, the effects on staff were measured through self-reporting, making it difficult to exclude the Hawthorne effect. Fourth, during the sustainability phase coinciding with the COVID-19 pandemic, visitor restrictions policy led to the inability to obtain consent from proxies, resulting in the loss of follow-up for residents. The dropout rate during this phase may have influenced the results. Finally, seasonal effects may have confounded the effect of the intervention. Future studies should consider these seasonal factors.

## Conclusions

Establishing the IPC program that addresses the WHO’s core components in an NH may have contributed to a reduction in the risk of respiratory infections among residents and improvements in infection control performance among staff. Applying the WHO’s core components in a low-resource setting, an LTCF, has demonstrated a positive impact. The findings of this study contribute to the design and development of effective IPC programs for NHs. Further studies that include control groups are necessary to validate the effects of the intervention.

## Electronic supplementary material

Below is the link to the electronic supplementary material.

Supplementary Material 1: Flowchart of participants in this study.

## Data Availability

The data collected and analysed during the current study are available from the corresponding author on reasonable request.

## Abbreviations

NHs: Nursing Homes
LTCFs: Long-Term Care Facilities
COVID-19: Coronavirus Disease 2019
IPC: Infection Prevention and Control
WHO: World Health Organization
RNs: Registered Nurses
HAI: Healthcare-Associated Infection
GI: Gastrointestinal
SST: Skin and Soft Tissue
HH: Hand Hygiene
SHEA: Society for Healthcare Epidemiology of America
APIC: Association for Professionals in Infection Control and Epidemiology
ANOVA: Analysis of Variance
OR: Odds Ratio
CI: Confidence Interval
ICLN: Infection Control Link Nurse
