# Supplementary figures and images for: Effects of establishing infection control program with core components of World Health Organization on reducing the risk of residents’ infections and improving staff infection control competency in a nursing home
Source: Antimicrob Resist Infect Control. 2024 Nov 14;13:136. doi: 10.1186/s13756-024-01492-4 (PMC11562619; doi:10.1186/s13756-024-01492-4)

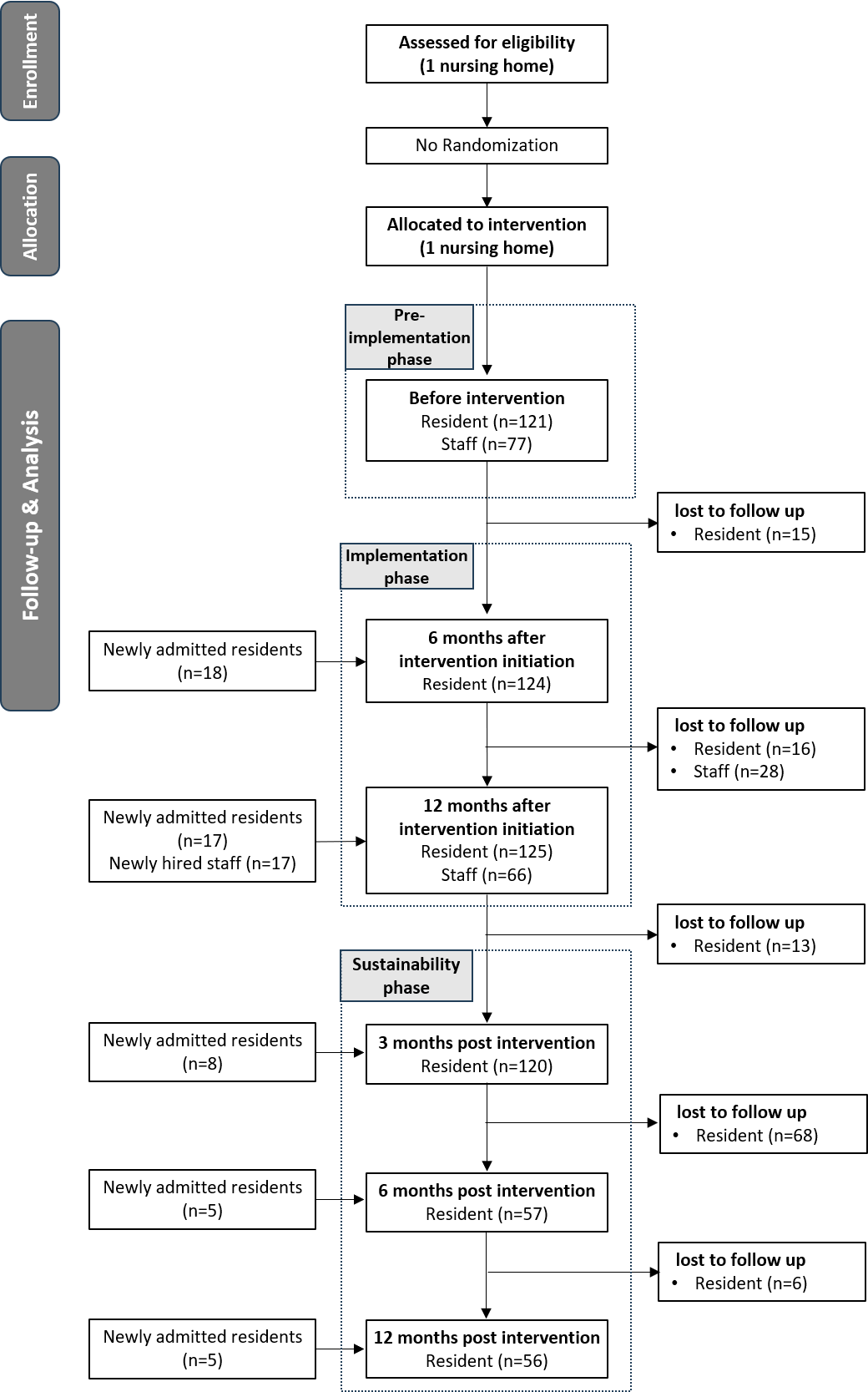

Supplement: Supplementary file 1 — Supplementary Material 1: Flowchart of participants in this study. [file 13756_2024_1492_MOESM1_ESM.png]
